# The implicit processing of categorical and dimensional strategies: an fMRI study of facial emotion perception

**DOI:** 10.3389/fnhum.2013.00551

**Published:** 2013-09-26

**Authors:** Yoshi-Taka Matsuda, Tomomi Fujimura, Kentaro Katahira, Masato Okada, Kenichi Ueno, Kang Cheng, Kazuo Okanoya

**Affiliations:** ^1^Okanoya Emotional Information Project, Exploratory Research for Advanced Technology (ERATO), Japan Science and Technology Agency (JST)Wako, Japan; ^2^Emotional Information Joint Research Laboratory, RIKEN Brain Science InstituteSaitama, Japan; ^3^Center for Baby Science, Doshisha UniversityKyoto, Japan; ^4^Department of Life Sciences, Graduate School of Arts and Sciences, The University of TokyoTokyo, Japan; ^5^Graduate School of Frontier Sciences, The University of TokyoKashiwa, Japan; ^6^Support Unit for Functional MRI, RIKEN Brain Science InstituteWako, Japan

**Keywords:** individual differences, fMRI, facial expressions, categorical processes, implicit

## Abstract

Our understanding of facial emotion perception has been dominated by two seemingly opposing theories: the categorical and dimensional theories. However, we have recently demonstrated that hybrid processing involving both categorical and dimensional perception can be induced in an implicit manner (Fujimura etal., 2012). The underlying neural mechanisms of this hybrid processing remain unknown. In this study, we tested the hypothesis that separate neural loci might intrinsically encode categorical and dimensional processing functions that serve as a basis for hybrid processing. We used functional magnetic resonance imaging to measure neural correlates while subjects passively viewed emotional faces and performed tasks that were unrelated to facial emotion processing. Activity in the right fusiform face area (FFA) increased in response to psychologically obvious emotions and decreased in response to ambiguous expressions, demonstrating the role of the FFA in categorical processing. The amygdala, insula and medial prefrontal cortex exhibited evidence of dimensional (linear) processing that correlated with physical changes in the emotional face stimuli. The occipital face area and superior temporal sulcus did not respond to these changes in the presented stimuli. Our results indicated that distinct neural loci process the physical and psychological aspects of facial emotion perception in a region-specific and implicit manner.

## INTRODUCTION

**FIGURE 1:**
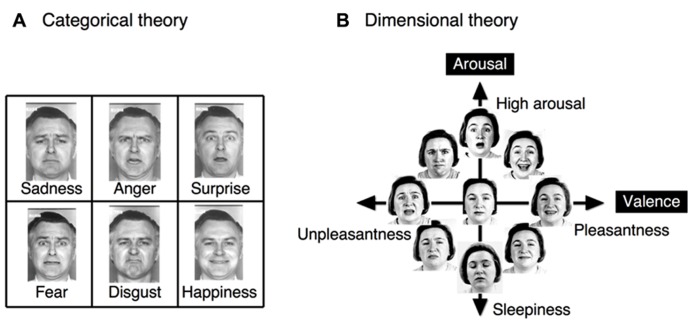
**The two different theories regarding facial emotion perception.** **(A)** The categorical theory proposes the existence of six basic, distinct and universal emotions, with clear boundaries separating emotional states. **(B)** The dimensional theory proposes the existence of the two fundamental dimensions of valence and arousal, which form an emotional space. In the dimensional theory, each emotional state may be continuously represented as a linear combination of values in these two dimensions. The original faces were drawn fromEkman and Friesen (1976), and Russell (1997).

Two seemingly opposing theories dominate the field of facial emotion perception: the categorical theory and the dimensional theory (**Figure 1**). The categorical theory posits the existence of six basic, distinct and universal emotions (Ekman et al., 1969; Ekman and Friesen, 1971, 1976; Ekman, 1992): happiness, anger, sadness, surprise, disgust, and fear (Tomkins and McCarter, 1964; Ekman and Friesen, 1971;Johnson-Laird and Oatley, 1992). The dimensional theory posits the existence of two fundamental dimensions of emotional space: valence and arousal (Russell, 1980; Russell and Bullock, 1985). Valence represents hedonic tone or position on a pleasantness–unpleasantness continuum, whereas arousal (Russell, 1980;Russell and Bullock, 1985) or tension (Schlosberg, 1954) refers to the level of energy of an affective experience. Evidence has been accumulated to support the occurrence of both categorical (Etcoff and Magee, 1992;Calder et al., 1996; Young et al., 1997) and dimensional (Katsikitis, 1997;Takehara and Suzuki, 1997, 2001) processing during facial emotion perception. In response to this apparent conflict, certain investigators have proposed a hybrid theory (Russell, 2003;Christie and Friedman, 2004;Panayiotou, 2008; cf.Rosch and Mervis, 1975) in which categorical and dimensional perception operate simultaneously to decode facial expressions. However, during the determination of facial emotions, it remains unknown which of these types of perception is relatively dominant and how these types of perceptions interact.

In a recent study (Fujimura et al., 2012), we used a stimulus set of morphed continua and two different sets of experimental instructions (detailing categorical vs. dimensional strategies) to investigate the relative dominance of categorical or dimensional perception during the decoding of facial expressions. Category boundaries were observed for not only identification and discrimination tasks, which require a categorical strategy, but also the Affect Grid task (Russell et al., 1989), which requires a dimensional strategy. Thus, our results indicate that categorical processing is dominant relative to dimensional processing and that categorical processing can be induced either explicitly or implicitly during facial emotion perception. Interestingly, this dominance of categorical processing was observed in the valence dimension but not the arousal dimension (Fujimura et al., 2012, Exp. 1). Despite this psychological evidence for the dominance of categorical processing, the neural mechanisms underlying this phenomenon remain unclear.

Facial emotion perception has been most frequently studied through the use of neuroimaging as a component of a general face perception study; this approach tends to indicate that a distributed neural system mediates both face processing and other types of cognitive processing (Sergent et al., 1992;Courtney et al., 1996;Haxby et al., 2000;Ishai et al., 2004, 2005;Calder and Young, 2005;Atkinson and Adolphs, 2011). The cortical network for face perception includes the inferior occipital gyrus (IOG) and lateral fusiform gyrus (FG;Kanwisher et al., 1997;Grill-Spector et al., 2004;Rotshtein et al., 2005); the superior temporal sulcus (STS;Puce et al., 1998;Hoffman and Haxby, 2000;Calder et al., 2007); the amygdala and insula (Breiter et al., 1996;Morris et al., 1996;Phillips et al., 1997;Vuilleumier et al., 2001;Ishai et al., 2004); the inferior frontal gyrus (IFG;Leveroni et al., 2000;Ishai et al., 2000); and the nucleus accumbens and orbitofrontal cortex (OFC;Aharon et al., 2001;O’Doherty et al., 2003;Kranz and Ishai, 2006). This distributed representation of face processing may reflect the fact that although the recognition of facial identity is based on invariant facial features, animated aspects of the face, such as speech-related movement and emotional expression, contribute to social communication (Ishai, 2008). This idea of a neural dichotomy between perceptions of facial identity and expressions within the “face network” represents an influential model in the field (Haxby et al., 2000); however, this proposal remains debated, as it has been demonstrated that certain brain regions are involved in perceiving both facial identities and expressions (Gorno-Tempini et al., 2001;Vuilleumier et al., 2001;Kaufmann and Schweinberger, 2004;Winston et al., 2004;Calder and Young, 2005;Ganel et al., 2005;Ishai et al., 2005;Fox and Barton, 2007;Palermo and Rhodes, 2007;Fox et al., 2008). However, to an extent, there exists consensus that specific neural processing mechanisms are associated with particular emotional facial expressions; for instance, disgust is processed in the insula, whereas fear is processed in the amygdala (Fusar-Poli et al., 2009). Three studies have utilized morphed faces to investigate the neural representation of categorical processing in facial emotion perception (Furl et al., 2007;Fox et al., 2009b;Harris et al., 2012); however, these studies have reported different neural bases for this phenomenon.Furl et al. (2007) used adaptation-induced after effects to identify the neural basis of categorical perception in the anterior temporal lobe. In contrast, Fox et al. (2009b) andHarris et al. (2012) employed a functional magnetic resonance imaging (fMRI) adaptation method to address this topic, although these fMRI studies produced inconsistent results. In particular, Fox et al. (2009b) found that the fusiform face area (FFA) and STS were responsive to the categorical differences in facial emotion continua, whereasHarris et al. (2012) demonstrated that categorical processing occurred in the amygdala. Similarly, studies of dimensional processing in facial emotion perception have provided inconsistent findings regarding the neural bases of this type of processing (Gerber et al., 2008;Fox et al., 2009b;Harris et al., 2012).

In this study, we sought to disentangle this question using our facial emotion stimulus sets, which were designed to simultaneously investigate not only categorical and dimensional processing but also valence and arousal, the two fundamental dimensions in emotional space (Fujimura et al., 2012). Furthermore, we sought to investigate which loci within the face network underlie the implicit categorical processing that we previously determined was dominant over dimensional processing in the valence dimension but not the arousal dimension.

We hypothesized that in emotional perception, categorical processing (i.e., the processing of psychological changes) and dimensional processing (i.e., the processing of physical changes) are encoded by separate neural loci of the face network. In particular, we hypothesized that there exists a functional dichotomy between the cortical and subcortical systems of the face network; in other words, this dichotomy exists between psychological processing in the cortical system and physical processing in the subcortical/limbic system. To test this hypothesis, we performed experiments using fMRI. Subjects were asked to perform irrelevant tasks to generate passive and implicit viewing of each face stimulus. Each stimulus was randomly presented from two morphed continua, which were created using the physical features of emotional faces (in particular, the happiness–fear continuum and the anger–disgust continuum were employed to examine the valence and arousal dimensions, respectively). We also asked subjects to perform psychological experiments that we have previously described (Fujimura et al., 2012), allowing us to identify individual differences in the categorical boundaries of the morphed continua. Individual categorical boundaries were then utilized to realign the response curves obtained from each subject’s blood oxygen level-dependent (BOLD) signal.

## MATERIALS AND METHODS

### STUDY PARTICIPANTS

A total of 22 Japanese adults participated in this study (12 males and 10 females; the mean ± SD of subjects’ ages was 27.7 ± 5.3 years). All of the subjects were right-handed and neurologically normal. Each subject provided written informed consent in accordance with procedures approved by the RIKEN Brain Science Institute Ethics Committee and Functional MRI Safety and Ethics Committee (Wako, Japan). Data obtained from four additional subjects were excluded from the analysis due to excessive head movement (>1 mm) and/or the termination of the experiment at the subject’s request.

### FACIAL STIMULI

**FIGURE 2:**
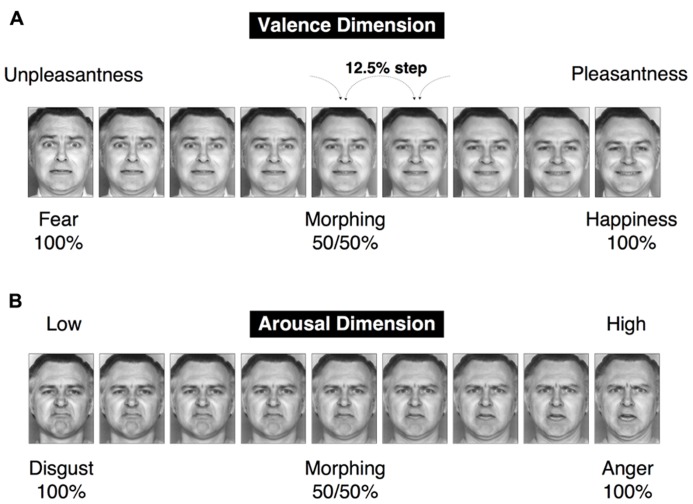
**A set of facial stimuli.** Two morphed continua were created, with a 12.5% step between a morphed image and the subsequent image in a continuum. The happiness–fear continuum represents change in the pleasantness–unpleasantness continuum (the valence dimension). The anger–disgust continuum represents change in the arousal–sleepiness continuum (the arousal dimension).

**FIGURE 3:**
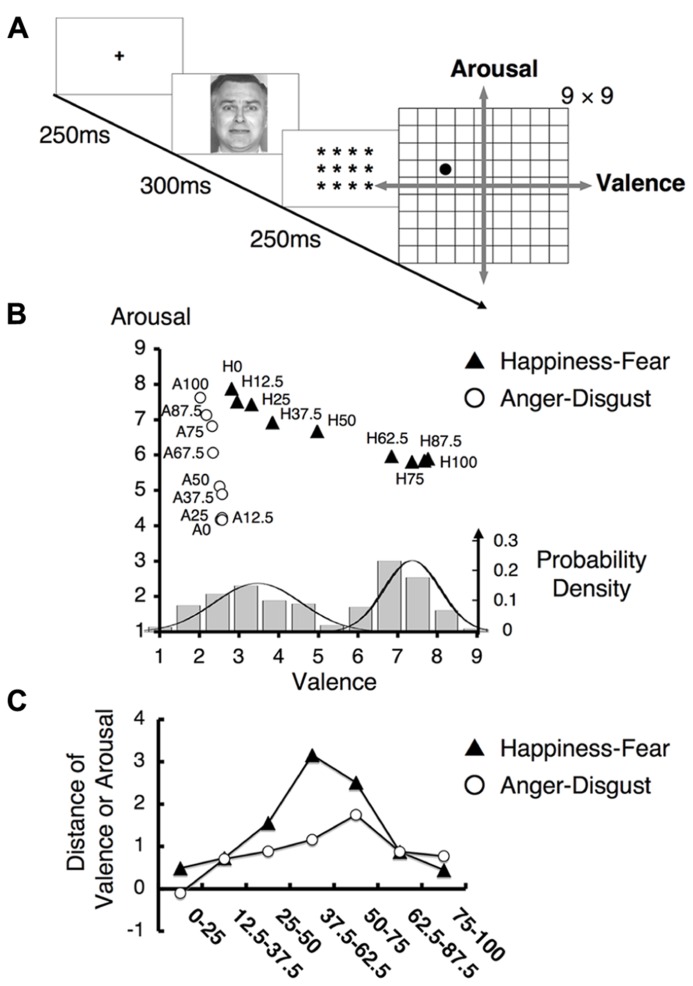
**Behavioral results for the Affect Grid task.** **(A)** The experimental design of the Affect Grid task. Subjects were asked to rate each facial emotion by selecting the appropriate location on a two-dimensional square grid representing the emotional space. This grid represents nine levels of both the valence and arousal dimensions; in particular, subjects rated each emotion from “1” = “unpleasant” to “9” = “pleasant” in the valence dimension and from “1” = “low” to “9” = “high” in the arousal dimension. **(B)** The mean ratings on the Affect Grid task. The labels “H12.5” and “A75” indicate the 12.5% happiness and 75% anger facial stimuli, respectively. The ratings for the happiness - fear continuum were nearly parallel to the valence axis, whereas the ratings for the anger–disgust continuum were approximately parallel to the arousal axis. (Bottom) The histogram indicates how frequently each facial stimulus on the happiness–fear continuum was assigned to each level of the valence scale on the grid representing the emotional space. The displayed data were obtained from every study participant and were averaged for each type of stimulus across all participants. The solid lines indicate normal distributions. **(C)** The mean differences in valence and arousal ratings between faces separated by two steps along the happiness–fear continuum and the anger–disgust continuum, respectively. The label “75–100” refers to the difference in valence (arousal) rating between the 75% happiness (anger) image and the 100% happiness (anger) image.

To simultaneously investigate both categorical and dimensional processing, facial stimuli were chosen from the six basic emotions, and a morphing technique was used to create intermediate stimuli among these emotions that could be interpreted from both categorical and dimensional perspectives. This set of visual stimuli, which was utilized in our prior research (Fujimura et al., 2012), was selected from the Facial Expressions of Emotion Stimuli and Tests (FEEST) devised byYoung et al. (2002). The stimuli utilized in our research were designed to dissociate the two fundamental dimensions of valence and arousal in the emotional space (**Figure 2**). In particular, our dimensional strategy attempted to dissociate the valence and arousal dimensions by creating two orthogonal continua that lay parallel to these two dimensions of the emotional space. To satisfy this constraint and the requirement that the emotional stimuli must be chosen from among the six basic emotions, fear and happiness were selected to create the valence-related continuum. This selection was motivated by the reasoning that these two emotions are the only pair of facial emotions that remain largely parallel to the two sides of the valence axis in the emotional space (with fear and happiness associated with negative and positive valences, respectively). Faces conveying disgust and anger were chosen to satisfy the aforementioned constraint for the arousal dimension (**Figure 1B**). However, there exist cross-cultural differences in facial emotion perception (e.g., Jack et al., 2012), particularly in the arousal dimension. Japanese individuals tend to perceive the disgust-anger continuum to be largely parallel to the arousal axis (Fujimura et al., 2012), whereas Caucasian individuals perceive the fear-sadness or surprise-sadness continua to be parallel to this axis (Russell, 1980). Expressions of fear and happiness were utilized as the endpoints for the creation of a morphed continuum in the valence dimension; in particular, the faces representing these two emotions were morphed to create seven intermediate images separated by steps of 12.5% (**Figure 2**). Using the same procedure, disgust and anger were employed to create a morphed continuum in the arousal dimension (**Figure 2**). We validated the stimuli to ensure their effectiveness for the study participants (see **Figure 3** in the section Results of this study).

We also created movie stimuli of the four emotional expressions (happiness, fear, anger, and disgust) and four mosaic faces. These stimuli were generated using the same faces of the same models (a male and a female) that were used for the main experimental sessions. These movie stimuli were used in a localizer task to effectively detect the “face network” in individual subjects by accounting for individual differences in brain structure (see the section fMRI DESIGN of this study). It has been reported that greater activation of the face network (e.g., the, FFA and IOG) is produced by emotional expression movies (which are known as “dynamic expressions”) than by static images of the same expressions (Kilts et al., 2003;LaBar et al., 2003;Sato et al., 2004;Fox et al., 2009a;Foley et al., 2012). Thus, we used these movies in a functional localizer task to define regions of interest (ROIs) in individual subjects (Fox et al., 2009a). Importantly, the same ROIs were obtained regardless of whether dynamic expressions or conventional static stimuli were used to define face-selective regions (Fox et al., 2009a;Pitcher et al., 2011a). This consistency was also reported in the ROIs defined using dynamic and static body postures as stimuli (Russell, 1997). We created the aforementioned movie stimuli in the following way. First, 24 intermediate images between the neutral (0%) and emotional (100%) expressions, separated by steps of 4%, were generated using computer-based morphing techniques. To create a movie clip, the resulting 26 images (the neutral image, the 24 intermediate images, and the final, 100% emotional image) were presented in succession. Each intermediate image was presented for 40 ms, and the first and last images were each presented for 520 ms; thus, the duration of each movie clip was 2,000 ms. It has previously been demonstrated that this presentation speed adequately reflects natural changes that occur in dynamic facial expressions of fear and happiness (Sato et al., 2004). A control condition was established by generating dynamic mosaic images from the same images used for the experimental condition (Sato et al., 2004). All of the aforementioned face images were arranged on a 12 × 18 grid and randomly reordered using a constant algorithm, causing each image to become unrecognizable as a face. A set of 26 of these mosaic images was serially presented as a movie clip. The presentation speed for these mosaic images was identical to the presentation speed utilized for the dynamic expression stimuli. These manipulations caused the dynamic mosaic images to be nearly equivalent to their corresponding original dynamic expression stimuli with respect to size, brightness and dynamic information.

### PSYCHOLOGICAL EXPERIMENT

Subjects were asked to perform a psychological experiment in a laboratory using the aforementioned facial stimuli. Events in the psychological experiment were controlled using a program written in Inquisit 3.0 (Millisecond, Seattle, USA) and implemented on a computer (Vostro 420, Dell) that used the Windows XP operating system (Microsoft). Static image stimuli were presented on a 19-inch LCD monitor (E1902S, Iiyama; 1024 × 768 pixels, 75 Hz refresh rate) and subtended a visual angle of approximately 10.0° × 7.3°. Participants rated the same facial stimuli using three types of tasks: the Affect Grid task, the identification task, and the ABX discrimination task. No time restrictions were applied. Four training trials were conducted prior to the performance of each task. The order of the three tasks was counterbalanced across the study participants.

#### The affect grid task (dimension task)

The 9 × 9 Affect Grid assesses affect along the dimensions of valence and arousal (Russell et al., 1989). Study participants were asked to rate the emotions expressed on the faces they viewed by utilizing a computer mouse to select the appropriate location on a two-dimensional square that represented the emotional space. Each trial began with the presentation of a fixation point for 250 ms, which was followed by the presentation of a blank screen for 250 ms and a facial stimulus for 300 ms. Following a mask stimulus of asterisks that lasted 250 ms, the Affect Grid was displayed until the participant responded. Each facial stimulus was presented twice in random order, producing a total of 72 trials that were divided into two blocks based on the two models who were utilized. The order of the blocks was counterbalanced across the participants.

#### The identification task (categorization task 1)

Participants were asked to identify depicted facial expressions by choosing between the two emotions on the endpoints of the continuum containing each depiction. For instance, participants were asked to identify the 87.5% happiness stimulus as either “happiness” or “fear.” Each trial began with the presentation of a fixation point for 250 ms, which was followed by the presentation of a blank screen for 250 ms and a facial stimulus for 300 ms. Subsequently, a mask consisting of a cluster of asterisks was displayed for 250 ms; two emotional words were then presented (“happiness? fear?” for the valence dimension and “anger? disgust?” for the arousal dimension), and participants were asked to select one of these two words by pressing the assigned button. Each face was presented eight times in random order, producing a total of 288 trials. The two models and two continua (happiness–fear and anger–disgust) of this study were used to divide these trials into four blocks. The order of the four blocks was randomly determined for each study participant.

#### The ABX discrimination task (categorization task 2)

The ABX discrimination task required participants to discriminate between faces from a continuum. Each trial began with the presentation of a fixation point for 250 ms, which was followed by the presentation of a blank screen for 250 ms and three successive images of faces. The first (A) and second (B) faces were presented for 300 ms each, and the third (X) face was presented for 1,000 ms. The blank intervals between the presentation of A and B, between the presentation of B and X, and after X were 250, 1,000, and 250 ms, respectively. Participants were asked to press a response button to indicate whether X matched A or B. In each trial, the stimuli A and B differed by two steps along one of the examined continua; thus, there was a 25% gap between the paired faces (e.g., happiness 100% and happiness 75%), and there were seven potential pairs for each continuum. The third face, X, was always identical to either A or B. Four presentation orders were possible: (ABX) = (ABA), (ABB), (BAA) or (BAB). The same order was presented twice for each pair, resulting in a total of 56 trials for each continuum. One block consisted of pairs from one continuum; thus, there were a total of four blocks. The order of the trials within a block and the blocks themselves were randomized across the study participants.

Chronologically, the psychological experiment was performed after the fMRI experiment (discussed below) to avoid any habituation/adaptation effects on the BOLD signal caused by excessive repetition of exposure to the same stimuli.

### fMRI DESIGN

Stimuli were displayed via a back-projection screen placed at the head of the scanner bore (Avotec Inc., Stuart, FL, USA; resolution: 800 × 600; refresh rate: 60 Hz), which was viewed by each subject via a mirror attached to the table near the subject’s head. A pair of plastic glasses was used to correct each subject’s vision to normal levels. All visual stimuli (260 × 360 pixels, gray images) were restricted to a maximum of 30° of visual angle. Manual responses were recorded using an MRI-compatible single-button box. An fMRI experiment consisted of three separate sessions (one functional localizer session and two main sessions).

In the localizer task of this study, dynamic expression stimuli (2,000 ms) were used to effectively define face network ROIs in individual subjects (Fox et al., 2009a;Pitcher et al., 2011a). Dynamic expressions and dynamic mosaics for the four types of examined expressions (happiness, fear, anger, and disgust) were randomly presented in turn, with each stimulus presented five times for each of the two model faces (a male and a female). Between trials, a central fixation cross was displayed for a jittered inter-trial interval of 3–6 s (mean = 4.5 s). In the localizer session, subjects were required to monitor a stream of dynamic expressions and dynamic mosaics and rapidly press a button when they detected a target “house” stimulus (100 ms), which was also randomly presented. This irrelevant task was performed to attract the attention of subjects, causing study participants to passively and implicitly view the presented facial expressions. Chronologically, the localizer session was performed after the main experiments (discussed below) to avoid any habituation/adaptation effects on the BOLD signal caused by excessive repetition of stimuli involving the same models’ faces.

In the main sessions, subjects were required to monitor a stream of “static” stimuli and rapidly press a button when they detected an upside-down “target” face. This irrelevant task was performed to attract the attention of subjects, causing study participants to passively and implicitly view the presented facial expressions. The targets were neutral faces that differed from the main stimuli but involved the same individuals. Each of the 18 expression images in the main stimulus set, which included the nine images of the morphed continuum for each of the two dimensions of emotional space (valence and arousal), was displayed (for 300 ms each) in quasi-random order. To avoid habituation/adaptation effects, there was no consecutive presentation of the same image in a raw. Between trials, a central fixation cross was displayed for a jittered inter-trial interval of 4–6 s (mean = 5 s). Different models (a male and a female with the same expressions) were used for each main session, and the order of the two main sessions was counterbalanced across the study participants. Each stimulus was presented six times for each model face.

### fMRI SCANNING PROCEDURE

Functional magnetic resonance imaging experiments were performed on a 4 T Agilent whole-body MRI system (Agilent Inc., Santa Clara, CA, USA) with a circularly polarized quadrature birdcage radio frequency coil as a transmitter and four-channel receiver surface coils as receivers (Nova Medical Inc., Wilmington, MA, USA). A total of 40 axial slices (24 cm field of view (FOV), 64 × 64 matrix, 3 mm thickness, 0 mm gap) with 30° forward rotation from the anterior commissure–posterior commissure (AC–PC) plane were acquired using a two-shot echo-planar imaging (EPI) pulse sequence [volume TR (repetition time) 4.4 s, TE (echo time) 25 ms, flip angle 78°] for the three functional runs (two main runs and one localizer run), each of which consisted of 156 volumes. After TSENSE (sensitivity encoding incorporating temporal filtering;Pruessmann et al., 1999;Kellman et al., 2001) reconstruction (with an acceleration factor of 2), the sampling frequency was doubled, causing the effective volume TR to become 2.2 s. Prior to and between the functional runs, a set of high-resolution (1 mm^3^) and low-resolution (1.72 mm^3^) whole-brain anatomical images were acquired using a magnetization-prepared 3D FLASH (fast low-angle shot) pulse sequence.

### ANALYSES OF fMRI DATA

#### Preprocessing

After EPI image reconstruction, intensity alternation between the odd- and even-numbered volumes produced by TSENSE reconstruction was removed from each functional run (odd and even volumes were averaged on a voxel-by-voxel basis, and we calculated the multiplication factor between the volumes). Cardiac and respiratory fluctuations were also removed using a retrospective estimation and correction method that we have previously described (Chen et al., 2001). The data were then preprocessed and analyzed using the BrainVoyager QX software package (Brain Innovation, Maastricht, Netherlands).

Data from the two main runs were serially connected in the time course direction. Preprocessing included slice scan time correction (using sinc interpolation), linear trend removal, temporal high-pass filtering to remove low-frequency non-linear drifts (0.00505 Hz) and 3D motion correction to detect and correct for small head movements by spatially aligning all volumes to a target volume via rigid-body transformations. A relatively small spatial smoothing isotropic Gaussian kernel that was 4 mm at full-width half-maximum (FWHM) was applied to the resulting volumes to investigate finer structures in the subcortical regions (e.g., the amygdala subregions). This spatial smoothing was applied to each subject’s native space (as a default component of preprocessing with Brain Voyager QX). Functional images were then coregistered to the anatomical volume, using both position parameters obtained from the scanner and manual adjustments to achieve the optimal fit. We omitted data from the four subjects who exceeded 1 mm in head movement (as estimated by the motion correction algorithm) and/or performed the tasks with less than 90% accuracy. Data from the remaining 22 adult subjects were used for the following analyses.

#### Functional localizers

After the transformation of each subject’s registered functional images into Talairach space (Talairach and Tournoux, 1988), whole-brain activation maps were obtained using a standard voxel-wise general linear model (GLM) at the single-subject level. For the functional localizer task, regressors encoding the perceptual processing of the four types of dynamic expressions that were examined (fear, happiness, disgust, and anger) and four types of dynamic mosaics were convolved using a theoretical two-gamma hemodynamic response function (HRF) and regressed against the observed BOLD data. To determine individual face-network ROIs for each subject, we compared the activity associated with all dynamic expression stimuli with the activity associated with all dynamic mosaics. For each subject, flexible thresholds [from uncorrected *p* < 0.001 to *p* < 0.00001, cluster size >4 voxels (36 mm^3^) and í10 voxels (90 mm^3^)] were employed to assess the results of this comparison and identify and isolate cluster of face-sensitive voxels in ROIs, particularly for subcortical regions, such as amygdala subregions. We adjusted *p* value for each ROI in each subject, so that the cluster size of each ROI fit within the definition (5~10 voxels) as small as possible. The *t*-statistics produced by these comparisons were computed to detect activation levels in each ROI. The three face-related regions comprising the “core” system of face perception were defined in the following manner (Fox et al., 2009a). Face-related voxels located on the lateral temporal portion of the FG were designated as the FFA, whereas voxels located on the lateral surface of the IOG were designated as the occipital face area (OFA). Face-related voxels located on the posterior segment of the STS were designated as the posterior portion of the superior temporal sulcus (pSTS). In addition to these core face-processing ROIs, regions comprising the “extended” system of face perception were also defined. Face-related voxels within the amygdala and insula were identified, and face-related voxels within the medial region of the prefrontal cortex were designated as the medial prefrontal cortex (mPFC).

#### ROI analyses

We then applied GLM analysis to each ROI of each subject to extract the enhanced BOLD signals involved in the perceptual processing of each emotional image (which included 18 emotional expressions in total from the valence and arousal continua, irrespective of the model who was examined). We generated 18 multiple regressors by convolving a boxcar representation of each type of stimulus-presentation period with the HRF. We then extracted the parameter estimates from this subject-specific (i.e., first-level) analysis. The parameter estimate for each ROI was extracted from the mean of all voxels in the cluster. Subsequent statistical correlational analyses of the generated linear and *U*-shaped models were performed using SPSS (IBM Corporation, Chicago, USA). All reported results were subjected to Greenhouse-Geisser correction for non-sphericity. To analyze the effect of the experimental conditions on BOLD signals in each ROI of the face network, we performed two types of analysis: a stimulus-based analysis and a perception-based analysis. Because fMRI results during a face/object detection task have indicated that the measured effects were driven more strongly by a subject’s perception than by stimulus category (Grill-Spector et al., 2004), it is possible that categorical effects observed in this study may be influenced less by the morph characteristics of the image than by whether the subject perceives the image as a specific expression of emotion. In fact, our previous study demonstrated that individual variations were observed when subjects were asked to identify categorical boundaries in morphed continua of facial expressions (Fujimura et al., 2012). To address this issue, we first performed a stimulus-based analysis using our *a priori* stimuli of morphed continua and aligned 50:50 morphed images in the centers of the tested continua (“5” in the valence/arousal score). We then calculated the BOLD response averages to each valence/arousal score across subjects (producing the “Stimulus-based” results, see fMRI RESULTS). Our second analysis realigned the stimulus images based on each subject’s category boundary, which was determined by the behavioral results from the categorization tasks (**Table 1**). We performed this realignment operation by implementing a parallel shift of each individual’s response curve from the stimulus-based analysis that adjusted subjective category boundaries to the center point for each dimension (“5” in the valence/arousal score). We then calculated the BOLD response averages to each valence/arousal score across subjects (producing the “Perception-based” results, see fMRI RESULTS).

**Table 1:** Individual category boundaries for each subject.

Subject	Category boundary (1–9)	
	Valence dimension	Arousal dimension
	(happiness–fear continuum)	(anger–disgust continuum)
ID 1	5	5
ID 2	5	6
ID 3	4	5
ID 4	5	6
ID 5	6	6
ID 6	5	5
ID 7	5	6
ID 8	5	6
ID 9	5	5
ID 10	6	6
ID 11	5	6
ID 121	5	6
ID 13	5	5
ID 14	6	6
ID 15	6	6
ID 16	5	5
ID 17	6	5
ID 18	6	6
ID 19	6	6
ID 20	6	6
ID 21	5	6
ID 22	8	5

*The individual category boundaries for each subject were defined as the facial stimulus with an identification rate closest to 50%. Each number corresponded to a morphed stimulus; as indicated in ***Figure 2***, these stimuli ranged from “1” = “0% happiness” to “9” = “100% happiness” in the valence dimension and from “1” = “0% anger” to “9” = “100% anger” in the arousal dimension. “5” denotes faces with a 50% contribution from each of the two opposing emotions of a continuum.*

A second-stage random-effect analysis (RFX) was performed solely to determine the coordinates of group-wise face network ROIs (uncorrected *p* < 0.001), which are indicated in **Table 2**.

**Table 2:** The ROIs defined by the functional localizer task of facial emotion perception.

Region-of-interest	*t*-value of peak voxel	Talairach coordinates		
		*x*	*y*	*z*
FFA (R)	4.82 ± 0.31	41 ± 7	-50 ± 9	-18 ± 6
OFA (R)	5.13 ± 0.47	44 ± 8	-65 ± 11	-11 ± 8
Amygdala (L)	4.47 ± 0.14	-25 ± 6	-2 ± 2	-12 ± 4
Insula (L)	4.86 ± 0.28	-31 ± 6	6 ± 8	-9 ± 7
mPFC	5.10 ± 0.41	-2 ± 2	55 ± 10	18 ± 8
pSTS (R)	5.51 ± 0.36	40 ± 8	-50 ± 11	3 ± 5

*The data reported above represent the average results from the functional localizer. The *t*-value of the peak voxel and the Talairach coordinates are indicated for each ROI (and expressed in terms of mean ± SD). The mean Talairach coordinates are reported solely to facilitate comparisons with the findings of other studies. FFA, fusiform face area; OFA, occipital face area; mPFC, medial prefrontal cortex; pSTS, posterior portion of the superior temporal sulcus; L, left hemisphere; R, right hemisphere.*

#### Validation with subject-wise analysis

We considered the issue that dimensional processing could be reflected in the linear relationships observed between BOLD signals and degrees of morphing. To analyze group-wise profiles, we calculated the *R*-values (the Pearson product-moment correlation coefficients) between the ordered integers 1 through 9 and the average BOLD signals across subjects for the nine stimuli along each dimension of the emotional space (valence or arousal, see fMRI RESULTS). We confirmed the group-wise profiles by performing a validation using the power of the random variable (the subjects) in the following manner. First, for each ROI and each subject, we calculated the Pearson *R*-value, which was subsequently converted to a *z*-value using Fisher’s *z*-transform. For each ROI, the 22 *z*-values from the study subjects were subjected to a one-sample *t*-test against zero. The average correction value (averaging the *R*-values across subjects) and the *t*-values with their associated *p*-values have been reported in **Table 3**.

**Table 3:** Analyses of dimensional (linear) processing in each ROI, after subject-wise validation.

ROI	Valence dimension						Arousal dimension					
	Stimulus-based			Perception-based			Stimulus-based			Perception-based		
	Ave. *R*	*t*_21_	*p*	Ave. *R*	*t*_21_	*p*	Ave. *R*	*t*_21_	*p*	Ave. *R*	*t*_21_	*p*
FFA (R)	-0.18	-2.03	0.06	-0.11	-0.93	0.36	0.10	1.68	0.11	0.13	2.08	<0.05*
Insula (L)	-0.15	-2.09	<0.05*	-0.13	-1.22	0.24	0.07	0.99	0.33	0.18	2.76	<0.05*
Amygdala (L)	-0.14	-2.11	<0.05*	-0.10	-1.26	0.22	0.06	0.51	0.62	0.08	0.77	0.45
mPFC	0.19	3.18	<0.01**	0.23	3.89	<0.001***	-0.03	-0.47	0.65	-0.01	-0.07	0.95
OFA (R)	-0.06	-0.79	0.44	-0.02	-0.21	0.84	0.12	1.58	0.13	0.10	1.30	0.21
pSTS (R)	0.02	0.41	0.68	0.01	0.11	0.92	-0.08	-0.84	0.41	-0.07	-0.77	0.45

The group-wise profile for dimensional processing in each ROI (**Figures 6** and **7**, as well as **Table 2**) was validated after the subject-wise validation (see the section Methods of this study). Average correction values (Ave. R, representing the mean of the R values from the 22 study subjects) and t-values with associated p-values are reported. *, ** and *** denote statistically significant correlations with p-values of less than 0.05, 0.01 and 0.001, respectively.

## RESULTS

### BEHAVIORAL RESULTS

We first confirmed that our stimuli affected the subjects of this study in a manner consistent with the effects observed in our previous study (Fujimura et al., 2012). In particular, we administered the Affect Grid task (**Figure 3A**), which used a dimensional strategy to confirm our previous findings regarding hybrid categorical and dimensional processing (Fujimura et al., 2012).

The mean scores on the Affect Grid task are illustrated in **Figure 3B**. Ratings of facial stimuli along the happiness–fear and anger–disgust continua shifted in accordance with changes in the valence or arousal dimensions, respectively, and were consistent with physical changes in the stimuli. These results indicated the existence of dimensional perception of the tested stimuli. The happiness–fear and anger–disgust continua were nearly orthogonal and remained relatively parallel to the valence and arousal dimensions, respectively, in the emotional space (**Figure 3B**). Although the happiness–fear continuum appeared to demonstrate a slight linear increase in arousal ratings as stimuli shifted from happiness to fear, this increase produced no significant difference between happiness and fear with respect to arousal ratings.

Notably, ratings on the happiness–fear continuum appeared to demonstrate a gap between the 62.5 and 50% stimuli, indicating the likelihood of a category boundary between these stimuli. However, no clear boundary was observed for the anger–disgust continuum; these findings were consistent with the results of our prior investigation (Fujimura et al., 2012). To visualize data distributions for the Affect Grid task, a frequency histogram for the happiness–fear continuum is provided in **Figure 3B** (bottom), indicating the ratings of the tested facial stimuli along the valence dimension. The rating data for each morphed stimulus were averaged for each subject. A Gaussian mixture model (McLachlan and Basford, 1988), which uses probability models to account for clustering in data distributions, was utilized for analysis. The Bayesian information criterion (BIC) was used to evaluate the fitness of the model; smaller BIC values indicate greater model suitability. We found two normal distributions in the data for the happiness–fear continuum (single Gaussian distribution: BIC = 971.07; two Gaussian distributions: BIC = 881.92). Thus, this study confirmed our previously reported finding (Fujimura et al., 2012) that valence may be divided into two clusters of rating scores; in other words, the study results support the existence of implicit categorical processing of emotion perceptions. To verify the occurrence of categorical perception in the presence of ratings based on continua in emotional space, we calculated the difference in valence or arousal ratings between facial stimuli differing by two steps on each examined continuum. The mean difference in valence rating between the elements of each pair of facial stimuli on the happiness–fear continuum and the mean difference in arousal rating between the elements of each pair of facial stimuli on the anger–disgust continuum are depicted in **Figure 3C**. We conducted an analysis identical to the approach used in the ABX discrimination task to confirm that the largest difference in valence rating among stimulus pairs on the happiness–fear continuum occurred between the stimuli with 67.5 and 37.5% happiness. This maximum difference in valence was compared with the combined mean valence differences of all other stimulus pairs on the happinessfear continuum, and a *t*-test demonstrated that the difference in valence between the 62.5 and 37.5% happiness images was significantly greater than the average valence difference between these other stimulus pairs (*t*_21_ = 7.20, *p* < 0.001). These results confirmed the validity of our stimuli and reproduced our previous findings regarding hybrid categorical and dimensional processing within the dimensional strategy (Fujimura et al., 2012).

We then performed categorization tasks to identify individual category boundaries for each subject, which were used in the *post hoc *analysis to realign individual fMRI data (to the “Perception-based analysis” results, as described in the section Methods of this study).

**FIGURE 4:**
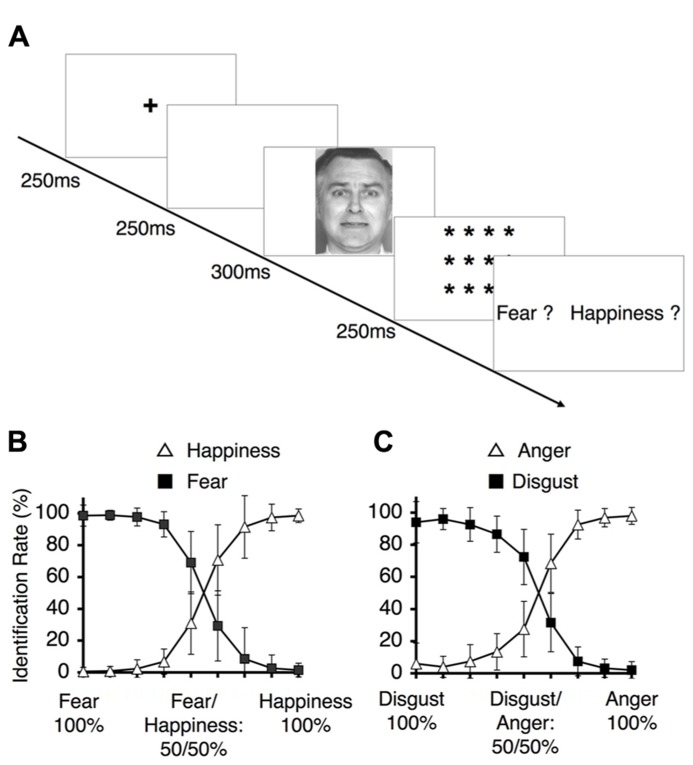
**Behavioral results for the identification task.** **(A)** The experimental design for the identification task. Subjects were asked to identify a depicted facial expression by choosing between the two emotions at the endpoints of the continuum to which the depiction belonged (i.e., fear vs*.* happiness, or disgust vs*.* anger). **(B)** The identification rates for the happiness-fear continuum. These rates indicate the frequencies at which happiness and fear were identified for the morphed faces depicted in **Figure 2A**. **(C)** The identification rates for the anger–disgust continuum. These rates indicate the frequencies at which anger or disgust were identified for each of the morphed faces depicted in **Figure 2B** (as indicated by the labels along the *x*-axis).

**FIGURE 5:**
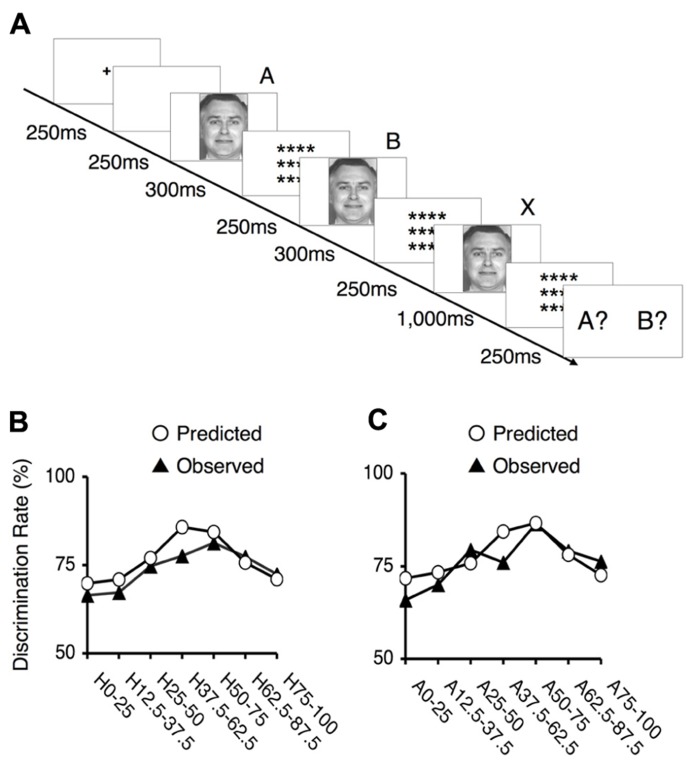
**Behavioral results for the ABX task.** **(A)** The experimental design of the ABX task. Subjects were asked to indicate whether the third face “X” matched the initial face “A” or the second face “B”. In each trial, the facial stimuli A and B differed by two steps on one of the examined continua; thus, there was always a 25% gap between this pair of faces (e.g., 100% happiness and 75% happiness), and seven potential pairs existed along each continuum. The third face, “X,” was always identical to either “A” or “B.” **(B)** The mean values of the observed discrimination rates and the predicted data for the happiness - fear continuum. The depicted rates were based on the frequency of correct responses in the ABX discrimination task. The labels indicate which pair of facial stimuli correspond to each point. For example, “H25–50” indicates a trial in which 25 and 50% happiness were presented as images A and B. **(C)** The mean values of the observed discrimination rates and the predicted data for the anger–disgust continuum. The labels indicate which pair of facial stimuli correspond to each point. For example, “A25-50” indicates a trial in which 25 and 50% anger were presented as images A and B.

The study subjects were asked to perform the two different categorization tasks of identification (**Figure 4A**) and ABX discrimination (**Figure 5A**). The results from the identification task are depicted in **Figures 4B,C**, which indicate the group-wise mean percentages for the two identified emotions on each continuum (happiness or fear in Figure 4B and anger or disgust in **Figure 4C**). Visual inspection of these figures reveals that the identification rates exhibit sigmoidal non-linear distributions, indicating the presence of an abrupt category shift within each continuum. On the happiness–fear continuum, the category boundary between happiness and fear appeared to be located between the stimuli with 62.5 and 50% happiness. The identification rates for the anger–disgust continuum also exhibited patterns similar to the patterns observed for the happiness–fear continuum.

To assess the occurrence of categorical perception, we applied a method used in previous studies (Calder et al., 1996;Young et al., 1997). First, we predicted subjects’ performance in the ABX discrimination task based on identification and ABX discrimination data. This approach assumes that two factors determine the ability to discriminate between two facial expressions: the physical differences between pairs of facial stimuli, independent of the expressions involved, and contributions from categorical perceptions of facial expressions. To estimate the first of these two factors, we utilized the mean of the discrimination rates for the pairs at the endpoints of each continuum. Categorical perception did not significantly contribute to these results because these stimuli were similar to prototypical facial expressions. To assess the second factor, we calculated the differences between the identification rates for the two relevant stimuli in each pair and multiplied this difference by 0.25 (a constant). By totaling the estimates for these two factors, we obtained performance predictions for the discrimination task. If these predicted values correlated with the observed ABX discrimination data, we could conclude that categorical perception occurred within the examined continuum.

The predicted rates of correct responses and the mean actual rates of correct responses for the discrimination task are indicated in **Figures 5B,C**. The observed and predicted curves appear to largely coincide, and the correlations between the observed and predicted results were significant for each continuum (for the happiness–fear continuum: *R* = 0.85, *t*_5_ = 3.56, *p* < 0.05; for the anger–disgust continuum: *R* = 0.76, *t*_5_ = 2.58, *p* < 0.05). Thus, categorical perception contributed to the observed responses to the facial stimuli within each continuum. If categorical perception occurs for each continuum, participants should more readily discriminate between a pair of facial stimuli that are on opposite sides of a categorical boundary than between a pair of facial stimuli that fall within the same category. To confirm this hypothesis, the peak correct discrimination rate for each continuum was compared with the mean of the correct discrimination rates for all other stimulus pairs along the continuum in question. A *t*-test revealed that the correct discrimination rate for the stimulus pair involving images of 75 and 50% happiness was significantly higher than the correct discrimination rates for other pairs along the happiness–fear continuum (*t*_21_ = 3.34, *p* < 0.01). On the anger–disgust continuum, significantly superior discrimination performance was observed for the stimulus pair involving images of 75 and 50% anger than for the other examined pairs (*t*_21_ = 4.67, *p* < 0.001). These results were consistent with our previous reports that 62.5% happiness and 62.5% anger may represent group-wise means that constitute category boundaries for the happiness–fear and anger–disgust continua, respectively, (Fujimura et al., 2012). Individual category boundaries for each subject were defined as the facial stimulus level with an identification rate that was closest to 50% (**Table 1**). If multiple facial stimuli exhibited identification rates that were approximately equivalent relative to this standard, the facial stimulus closest to the center of the continuum (that is, the stimulus that was closest to 50% for each of the two opposing emotions of a continuum) was defined as the category boundary for the subject in question.

### fMRI RESULTS

Given that our psychological results exhibited hybrid categorical and dimensional processing in the valence dimension but not the arousal dimension (**Figure 3**), we sought to identify the neural basis of this phenomenon. To test our hypothesis that categorical and dimensional processing are intrinsically encoded by separate neural loci, we induced study subjects to passively view each emotional stimulus by asking these subjects to perform irrelevant tasks. The fMRI experiments involving passive viewing of the tested emotional stimuli were conducted prior to the aforementioned behavioral experiments to minimize any neural adaptation effects that might be induced by the repetitive presentation of the same stimuli in both sets of experiments. We obtained data from limited ROIs defined by a subsequent functional localizer task (as described in the section Methods of this study).

In accordance with the findings of numerous other reports, the functional localizer experiment conducted in this study revealed the existence of a distributed neural network for emotional face processing (**Table 2**). We depicted the response properties along the happiness–fear and anger–disgust continua in each ROI and classified the observed responses into three different groups with respect to facial emotion processing: categorical, dimensional (linear) and constant (uniform) processing. The valence and arousal dimensions were separately analyzed using the happiness–fear and anger–disgust continua, respectively.

#### Categorical processing in the valence dimension

**FIGURE 6:**
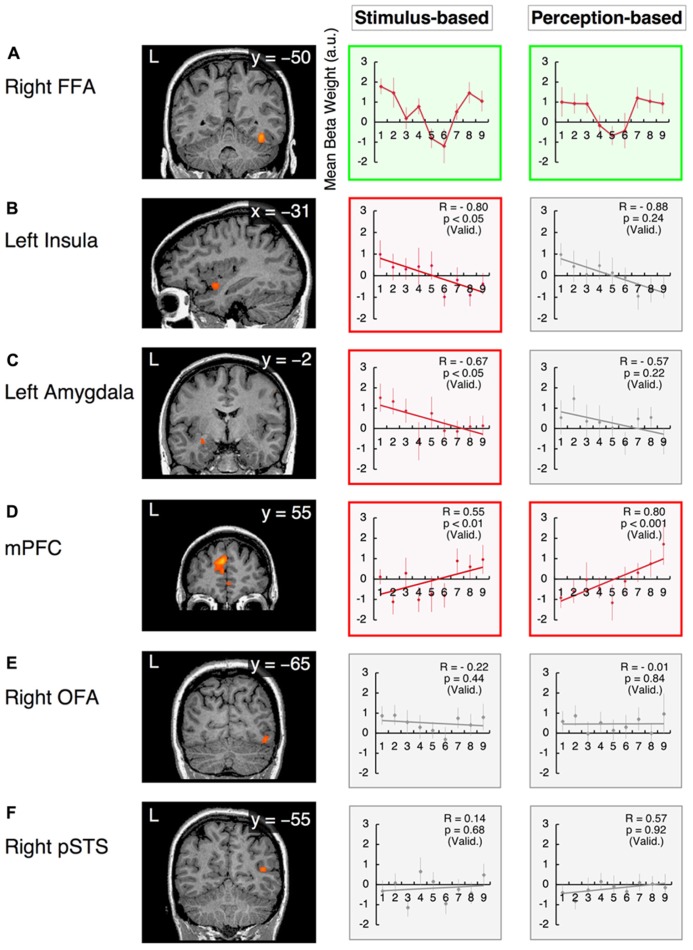
**Functional magnetic resonance imaging results in the valence dimension.** The x-values represent morphed faces on the happiness - fear continuum depicted in **Figure 2A**, ranging from “1” = “0% happiness” to “9” = “100% happiness.” The *y*-values represent the BOLD signals [with arbitrary units (a.u.)] obtained from each ROI. Two graphs are provided for each ROI to indicate the results from the stimulus-based and perception-based analyses; in the latter analysis, each individual’s fMRI data are realigned in accordance with the individual’s behavioral data with respect to category boundaries (**Table 1**). The graphs in green squares represent *U*-shaped or categorical processing that differs from linear or constant processing. Graphs in red squares represent dimensional (linear) processing with statistically significant correlations after the subject-wise validation (**Table 3**). Graphs in gray squares represent constant processing without statistically significant correlations. Error bars denote SEM. R: the group-wise correlation coefficient. p (Valid.): the probability after the subject-wise validation (**Table 3**). **(A)** The right fusiform face area (FFA); **(B)** the left insula; **(C)** the left amygdala; **(D)** the mPFC; **(E)** the right OFA; and **(F)** the posterior STS (pSTS) in the right hemisphere.

The right FFA exhibited non-linear processing instead of a linear or uniform response in the happiness–fear continuum (**Figure 6A**, “Stimulus-based” results); as responses in the right FFA did not demonstrate a statistically linear correlation with the valence scores of facial emotions after the subject-wise validation (group-wise *R* = -0.19; after the subject-wise validation: *t*_21_ = -2.03, *p* = 0.06; see the section Methods of this study for additional details). Visual inspection indicates a *U*-shaped response curve in this ROI, with greater signal for both the happier and more fearful faces but lower signal for faces that are approximately evenly divided between these two emotions. This phenomenon suggests that the right FFA contributes to categorical processing via the detection of unambiguous faces (at both ends of the happiness–fear continuum) but filters out ambiguous faces near the category boundary. To confirm this abrupt change in response property for approximately evenly divided faces, we statistically compared the average responses of the right FFA to the three faces on each end of the happiness–fear continuum (i.e., the 100-0, 87.5-12.5 and 75-25 faces at both the happiness and fear ends of this continuum) with the three faces at the center of the continuum (the 37.5-62.5, 50-50 and 62.5-37.5 faces). This statistical comparison confirmed that the right FFA exhibited significantly different responses to the faces at the ends of the happiness–fear continuum than to the faces in the center of this continuum (*t*_21_ = 2.51, *p* < 0.05). The group-wise response property of categorical processing became more evident when the fMRI data from each study subject were realigned in accordance with the subject’s category boundary, as determined by the behavioral results from the categorization tasks (**Table 1** and the “Perception-based” results in **Figure 6A**). We performed this realignment by implementing a parallel translation of each individual’s response curve that adjusted the category boundary to the center for each dimension (as represented by a valence or arousal score of “5”). For the study participants, neural responses in the right FFA to faces that were perceived to be unambiguous (the three faces at each end of a subject’s perceived happiness–fear continuum) were not only relatively constant but also significantly greater than the neural responses in this ROI for faces that were perceived to be ambiguous (the central three faces of a subject’s perceived happiness–fear continuum). In particular, in the comparison of average responses for the three faces on each end of the continuum with average responses for the three central faces, *t*_21_ = 2.17, with *p* < 0.05. This response property of categorical processing differed from the typical sigmoidal curve obtained in the behavioral results (**Figure 4B**). Instead, the observed response, which appears to approximate the first derivative of a sigmoid function rather than a simple *U*-shaped curve, exhibited categorical processing with abrupt changes in response near a perception-based categorical boundary and with constant processing near both ends. We statistically compared response curves of the FFA between stimulus-based and perception-based analyses to find a better fit by the first derivative of sigmoid function. There was a trend that a perception-based response curve was better fitted by the first derivative of sigmoid function (*t*_21_ = 1.89, *p* = 0.07). This result indicated that realignment of each individual’s response curve by using individual’s category boundary was essential for the better fit, and hence implicated that the FFA exhibited categorical processing. These results indicated that the FFA not only engages in categorical processing but also detects an individual’s categorical boundary in the valence dimension and decreases its response accordingly.

Although the observed atypical “*U*-shaped” curve for the BOLD response as a function of morphing may not constitute compelling evidence for categorical processing, the right FFA was the only region in the face network to exhibit categorical processing in the valence dimension that clearly differed from linear or constant processing (as discussed below).

In contrast, in the arousal dimension, the right FFA exhibited dimensional processing (as discussed below) rather than categorical processing. This phenomenon was consistent with our psychological results, which indicated that implicit categorical processing occurred in the valence dimension but not the arousal dimension. Thus, the right FFA is a candidate ROI for the neural basis of psychological processing in facial emotion perception (see the section Discussion of this study).

#### Dimensional (linear) processing

The response in the left insula exhibited a negative correlation with the valence scores of facial emotions (Figure 6B). Activity in this region increased in response to increasingly fearful, “unpleasant” faces but decreased in response to increasingly happy, “pleasant” faces. The linear response property of this ROI was statistically significant in stimulus-based analyses but not perception-based analyses (stimulus-based results: group-wise *R* = -0.80, with *t*_21_ = -2.09 and *p* < 0.05 after the subject-wise validation; perception-based results: group-wise *R* = -0.88, with *t*_21_ = -1.22 and *p* = 0.24 after the subject-wise validation; Figure 6B, **Table 3**). The left amygdala also exhibited a negative correlation with valence scores that was significant only in stimulus-based analyses (stimulus-based results: group-wise *R* = -0.67, with *t*_21_ = -2.11 and *p* < 0.05 after the subject-wise validation; perception-based results: group-wise *R* = -0.57, with *t*_21_ = -1.26 and *p* = 0.22 after the subject-wise validation; **Figure 6C, Table 3**). The mPFC exhibited a significant positive correlation with valence scores in both the stimulus-based and perception-based analyses (stimulus-based results: group-wise *R* = 0.55, with *t*_21_ = 3.18 and *p* < 0.01 after the subject-wise validation; perception-based results: group-wise *R* = 0.80, with *t*_21_ = 3.89 and *p* < 0.001 after the subject-wise validation; **Figure 6D, Table 3**).

**FIGURE 7:**
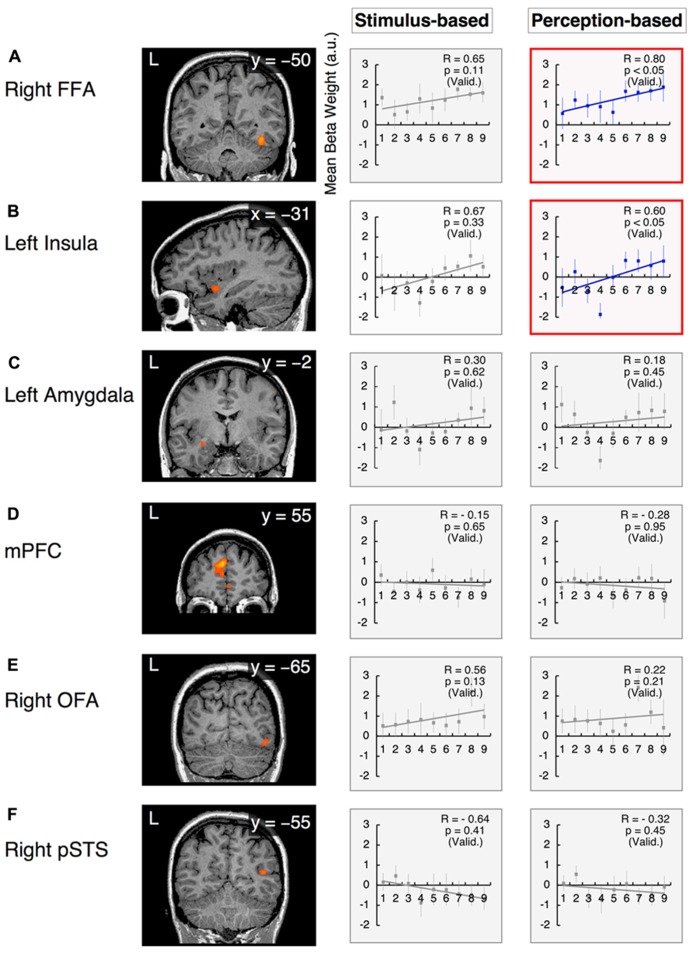
**Functional magnetic resonance imaging results in the arousal dimension.** The *x*-values represent morphed faces on the anger–disgust continuum depicted in **Figure 2B**, ranging from “1” = “0% anger” to “9” = “100% anger.” The *y*-axes represent the BOLD signals [with arbitrary units (a.u.)]. Two graphs are provided for each ROI to indicate the results from the stimulus-based and perception-based analyses. Graphs in red squares represent dimensional (linear) processing with statistically significant correlations after the subject-wise validation (**Table 3**). Graphs in gray squares represent constant processing without statistically significant correlations. Error bars denote SEM. R: the group-wise correlation coefficient. p (Valid.): probability after the subject-wise validation (**Table 3**). **(A)** The right FFA; **(B)** the left insula; **(C)** the left amygdala; **(D)** the mPFC; **(E)** the right OFA; and **(F)** the right pSTS.

However, in the arousal dimension, the right FFA exhibited a positive correlation with arousal scores of facial emotions, although this correlation was significant only in perception-based analyses (stimulus-based results: group-wise *R* = 0.65, with *t*_21_ = 1.68 and *p* = 0.11 after the subject-wise validation; perception-based results: group-wise *R* = 0.80, with *t*_21_ = 2.08 and *p* < 0.05 after the subject-wise validation; **Figure 7A, Table 3**). The left insula also exhibited a significant positive correlation with arousal scores in perception-based analyses but not stimulus-based analyses (stimulus-based results: group-wise *R* = 0.67, with *t*_21_ = 0.99 and *p* = 0.33 after the subject-wise validation; perception-based results: group-wise *R* = 0.60, with *t*_21_ = 2.76 and *p* < 0.05 after the subject-wise validation; **Figure 7B, Table 3**).

This linear-fit analysis appeared to render linear processing and categorical “sigmoid-curve” processing indistinguishable because both of these processing types should exhibit high correlation coefficients (monotonically increasing functions). This confounding problem may be inevitable given that we measured BOLD signals with large per-trial variability; furthermore, this issue may have been exacerbated in this investigation because we used a small number of trials (12 for each emotional face, regardless of the model involved) to prevent adaptation effects. Indeed, there were no statistical differences between sigmoidal- and linear-fit models to explain our neural response function (*t*_21_ < 1.29 and *p* > 0.21 for all ROIs). Thus, we only categorized the remarkable case of the right FFA, which involved a clear boundary as an instance of categorical processing (as discussed above).

We excluded the possibility that data from the aforementioned areas that demonstrated dimensional processing could also be explained by *U*-shaped categorical processing. For these areas, there were no statistically significant differences between the neural responses to unambiguous faces (the three faces at each end of a continuum) and the neural responses to ambiguous faces (the three faces in the center of the continuum in question) for either stimulus-based or perception-based analyses (in particular, for all cases, the comparison of the average responses of the three faces at each end of a continuum with the three faces at the center of the curriculum produced results of *t*_21_ < 1.70 and *p* > 0.1).

#### Constant processing

The left amygdala and mPFC demonstrated relatively constant (or uniform) processing in the arousal dimension in both stimulus-based and perception-based analyses [stimulus-based results for the left amygdala: group-wise *R* = 0.30, with *t*_21_ = 0.51 and *p* = 0.62 after the subject-wise validation; perception-based results for the left amygdala: group-wise *R* = 0.18, with *t*_21_ = 0.77 and *p* = 0.45 after the subject-wise validation (**Figure 7C** and **Table 3**); stimulus-based results for the mPFC: group-wise *R* = -0.15, with *t*_21_ = -0.47 and *p* = 0.65 after the subject-wise validation; perception-based results for the mPFC: group-wise *R* = -0.28, with *t*_21_ = -0.07 and *p* = 0.95 after the subject-wise validation (**Figure 7D** and **Table 3**)]. The right OFA and right pSTS also demonstrated constant processing in both the valence and arousal dimensions (for all cases, group-wise *R* < 0.65, with *p* > 0.2 after the subject-wise validation; **Figures 6E,F and 7E,F** as well as **Table 3**). However, the OFA and STS differed in activity levels. The right OFA exhibited significant activation relative to baseline levels (*t*_21_ = 2.68, *p* < 0.05), whereas no significant activation was observed for the STSs (for both the left and right STS, *t*_21_ < 0.36 and *p* > 0.70). Notably, we defined functional ROIs using dynamic-expression stimuli of emotional faces in an implicit manner (**Table 2**) but investigated categorical/dimensional processing using static facial images (as described in the section Methods of this study). Our results indicated that in the absence of explicit attention, the right OFA responds to both static and dynamic emotional face stimuli, whereas the right STS responds to dynamic but not static images.

We excluded the possibility that the data from areas that exhibited constant processing could be explained by U-shaped categorical processing. For these areas, there were no statistically significant differences between the neural responses to unambiguous faces (the three faces at each end of a continuum) and the neural responses to ambiguous faces (the three faces in the center of the continuum in question) for either stimulus-based or perception-based analyses (in particular, for all cases, the comparison of the average responses of the three faces at each end of a continuum with the three faces at the center of the curriculum produced results of *t*_21_ < 1.30 and *p* > 0.2).

## DISCUSSION

The current investigation is the first study to simultaneously investigate the implicit processing of both categorical and dimensional aspects of facial emotion perception. Our experimental design dissociates the two fundamental dimensions of valence and arousal in the emotional space by establishing two orthogonal continua of morphed stimuli that parallel these dimensions. Because the four endpoints of the morphed continua represented four basic emotions (happiness, fear, anger, and disgust), this design enabled the interpretation of morphed stimuli from both categorical and dimensional perspectives. The results obtained from an fMRI study involving the passive viewing of emotional stimuli along the examined continua confirmed our hypothesis that the implicit encoding of categorical and dimensional (linear) processing in separate neural loci serves as a basis for our psychological observations of hybrid processing. In the valence dimension (the happiness–fear continuum), the right FFA exhibited categorical processing, whereas the left insula, left amygdala and mPFC exhibited linear processing. However, in the arousal dimension (the anger–disgust continuum), the right FFA and left insula demonstrated linear processing, and none of the examined loci demonstrated clearly categorical processing. Notably, the right FFA was the only area to reflect our psychological results, which indicated that categorical processing occurred in the valence dimension and linear processing occurred in the arousal dimension in an implicit manner (**Figure 3**; Fujimura et al., 2012).

### CATEGORICAL PROCESSING IN THE VALENCE DIMENSION

Categorical processing in the right FFA became more obvious in the group-wise profile after the fMRI data from individual subjects were realigned to conduct perception-based analyses based on the category boundaries established for each subject from the behavioral results (**Figure 6A**). This phenomenon indicates that implicit categorical processing in the right FFA is likely to reflect the explicit behavioral strategy of categorical processing. Given that category boundaries vary by individual (**Table 1**), the right FFA may implicitly process subjective values of category boundaries in the facial continuum of the valence dimension. This conjecture is consistent with the known role of the FFA in processing psychological (i.e., subjective) rather than physical aspects of face stimuli. Moreover, in morphed face experiments involving individual identification tasks, Rotshtein et al. (2005) and Fox et al. (2009b) have determined that the FFA is sensitive to perceived similarity but not physical similarity. It has also been reported that psychological processing in the FFA exhibits the face-inversion effect, which involves a higher response to upright faces than to inverted faces; this difference has been correlated with behavioral effects across subjects (Yovel and Kanwisher, 2005). The findings from the current investigation, in combination with the aforementioned results from published studies and other accumulating evidence supporting the role of the FFA in emotion processing (for a review, seeFusar-Poli et al., 2009), suggest that the FFA processes not only the psychological (i.e., the categorical) aspects of face identification but also contributes to emotion recognition in face perception; this conjecture is consistent with previously reported results (Fox et al., 2009b). In this investigation, we have also confirmed that the implicit categorical processing of facial emotion perception occurs in the valence dimension (the happiness–fear continuum) but not the arousal dimension (the anger–disgust continuum). The right FFA was the only examined brain area to reflect our psychological results indicating the existence of categorical processing in the valence dimension and linear processing in the arousal dimension in an implicit manner (**Figure 3**; Fujimura et al., 2012).

### DIMENSIONAL PROCESSING IN THE VALENCE DIMENSION

Activity in the left insula and left amygdala was negatively correlated with the valence scores of facial emotions; thus, in these regions, increasingly unpleasant faces produced greater neural responses, whereas increasingly pleasant faces produced smaller neural responses (**Figures 6B,C**). Previous attempts to investigate how the response of the insula relates to the valence of a stimulus have generated conflicting results; one study has reported that these traits are negatively correlated (Cunningham et al., 2004), several investigations have stated that these traits are positively correlated (Heinzel et al., 2005; Posner et al., 2009), another study has claimed that both negative and positive correlations between these traits could be observed depending on which insula subregion is examined (Lewis et al., 2007), and one investigation has identified an inverted U-shaped relationship between stimulus valence and insula response (Viinikainen et al., 2010). The discrepancies among these findings might reflect the differing stimulus sets used in the aforementioned investigations. Although conventional stimulus sets [e.g., the International Affective Picture System (IAPS);Lang et al., 2005] span a wide range of emotional space, each stimulus within a conventional set consists of a completely different face or scene. Furthermore, in these stimulus sets, valence and dimensional scores tend to be correlated; in particular, both highly pleasant and highly unpleasant stimuli appear to elicit high arousal. Thus, valence or arousal scores in the emotional space cannot be systematically altered using these stimulus sets, rendering it difficult to functionally dissociate the valence and arousal dimensions in a research investigation. In contrast, our morphed stimulus set utilizes faces with the same identities and has been designed to enable either the valence or arousal score alone to change as the remaining parameter remains fixed. This stimulus set design can ideally dissociate the valence and arousal dimensions of the emotional space, resulting in data sets suitable for subsequent analysis. It has been demonstrated that the insula plays a role in a number of unpleasant affective states, such as anger (Damasio et al., 2000); disgust (Calder et al., 2000); physical pain and social distress (Eisenberger et al., 2003); empathy for others’ pain (Singer et al., 2004); and guilt (Shin et al., 2000; for a review, seeCraig, 2009). These studies support our results, which indicate a negative correlation between insula response and the valence scores of facial emotions. However, further studies are required to elucidate how insula activity relates to valence scores; in particular, these future investigations could examine each subregion of the insula and/or asymmetry between the left and right hemispheres (Craig, 2009).

Although it has been well documented that the amygdala is activated during the presentation of emotional stimuli (LaBar et al., 1995, 1998;Zald, 2003), the precise role of the amygdala in emotional processing and the direction of amygdala activation remain the subject of considerable controversy (for a review, seeCostafreda et al., 2008). For example, the amygdala has been implicated in mediating emotional responses not only to discrete emotions, such as fear (LaBar et al., 1995), but also to the properties of emotional cues, such as valence (Morris et al., 1998;Garavan et al., 2001;Killgore and Yurgelun-Todd, 2004;Fusar-Poli et al., 2009;Gerdes et al., 2010;Viinikainen et al., 2010), intensity (i.e., arousal;Anderson et al., 2003;Small et al., 2003;Glascher and Adolphs, 2003;Anders et al., 2004;Cunningham et al., 2004;Kensinger and Corkin, 2004;Kensinger and Schacter, 2006;Lewis et al., 2007;Gerber et al., 2008) or a combination of both valence and intensity (Anders et al., 2004;Kensinger and Corkin, 2004;Winston et al., 2005). The discrepancies among these findings might reflect the insufficient resolution of conventional fMRI for investigations of the human amygdaloid complex. In fact, recent studies have sought to dissociate the different functional subregions of the amygdala through the use of high-resolution fMRI (Davis et al., 2010;Gamer et al., 2010). Our findings from the current study indicating that the amygdala is preferentially involved in processing valence rather than arousal are consistent with the results of these recent studies, which have demonstrated greater activity in the lateral subregion of the amygdala in response to unpleasant faces than in response to pleasant faces (Davis et al., 2010;Gamer et al., 2010).

The mPFC exhibited positive correlations with valence scores for facial emotions, with increased responses to pleasant faces and decreased responses to unpleasant faces (**Figure 6D**, “Perception-based” results). The mPFC has a well-established role in emotional processing, particularly with respect to reward mechanisms (Knutson et al., 2003;Phillips et al., 2003, 2008;Sabatinelli et al., 2007). The mPFC is more consistently activated when a subject perceives or attempts to modulate a subjective feeling during the perception of an emotionally evocative stimulus than when these processes occur in response to an emotionally neutral stimulus (Lane et al., 1997;Reiman et al., 1997;Ochsner et al., 2002;Phan et al., 2002). Interestingly, the opposing correlations with valence observed in this study for mPFC activity (positively correlated with valence) and amygdala activity (negatively correlated with valence; **Figures 6C, D**) are consistent with the findings from animal models and human imaging studies, which indicate a reciprocal relationship between prefrontal and amygdala activities (Davidson, 2002;Quirk et al., 2003;Likhtik et al., 2005). Our findings suggest that higher levels of neural activity in the mPFC in response to stimuli with greater positive valences are accompanied by concurrent declines in amygdala activity. This result is consistent with the findings of previous studies of functional connectivity that have demonstrated inverse correlations between PFC activity and amygdala activity (Zald et al., 1998;Kim et al., 2003;Quirk et al., 2003). Several investigators have suggested that this reciprocal relationship may represent a regulatory or feedback system that serves to modulate and dampen affective responses that would otherwise be excessive (Garcia et al., 1999;Ochsner et al., 2002).

### DIMENSIONAL PROCESSING IN THE AROUSAL DIMENSION

The right FFA and left insula exhibited positive correlations with the arousal scores of facial emotions, with increased responses to faces with higher arousal levels (**Figures 7A,B**). Previous studies have also proposed that the arousal of facial emotions affects FFA activity (Glascher et al., 2004;Kanwisher and Yovel, 2006;Brassen et al., 2010), although our results extend these findings by suggesting that valence (pleasant–unpleasant) also affects FFA activity (as discussed above). We found that the right FFA differentially processes the arousal and valence dimensions, engaging in dimensional (linear) processing for arousal and categorical processing for valence (**Figures 6A** and **7A**). However, simple arousal effects alone cannot explain our FFA activity results. For example, the arousal score of the 100% disgust face (i.e., the 0% anger face) was lower than the arousal score of the 50% happiness face (i.e., the 50% fear face; **Figure 3B**); however, there was a greater FFA response to the 100% disgust face than to the 50% happiness face (*t*_22_ = 3.14, *p* < 0.01). This phenomenon indicate the integrated nature of arousal and valence processing in the right FFA, which enhances its activity in response to higher-arousal faces and filters out emotionally ambiguous faces in the valence dimension.

However, the left insula demonstrated linear processing in both the arousal and valence dimensions (**Figures 6A** and **7A**). In particular, enhanced left insula activity was observed for faces with greater arousal and greater unpleasantness. These results are consistent with previous reports demonstrating that negative words with higher arousal activated the middle subregion of the left insula (Lewis et al., 2007), which is located in the vicinity of the functional ROI that we identified in the left insula in the current investigation.

### CONSTANT PROCESSING IN THE VALENCE AND AROUSAL DIMENSIONS

The right OFA and right pSTS exhibited constant (or uniform) processing in both the valence and arousal dimensions (**Figures 6E,F** and **7E,F**). The right OFA is known to play an essential role in face recognition, as lesions in the right OFA induce prosopagnosia (Rossion et al., 2003;Sorger et al., 2007). It has also been suggested that the OFA preferentially represents certain facial components, including the eyes, nose, and mouth, at an early stage of visual perception (Pitcher et al., 2011b). In fact, it has been hypothesized that by representing these facial components, the OFA functions as the first stage in a hierarchical face perception network in which increasingly complex facial features are subsequently processed by higher face-selective cortical regions (Haxby et al., 2000). Consistent with these studies, our investigation demonstrated the constant activation of the right OFA to any morphed stimulus of emotional faces, irrespective of the valence and arousal scores of these faces. This phenomenon might reflect the fact that our stimuli involved the same facial identities (i.e., all of the examined emotional expressions utilized the same models); thus, the right OFA might respond similarly to the identical facial components within these stimuli.

However, the right pSTS demonstrated virtually no response to any stimulus in the valence and arousal dimensions when static facial expressions were presented in an implicit manner (**Figures 6F** and **7F**), in contrast to the results observed with explicit processing (Puce et al., 1998;Hoffman and Haxby, 2000;Calder et al., 2007). Interestingly, the right pSTS was responsive when dynamic facial expressions were used as functional localizer stimuli (**Table 2**). One possible explanation for this finding is that even in an implicit context, relative to the traits of static faces, the characteristics of dynamic faces might produce enhanced visual motion analysis by recruiting more attentional resources and inducing stronger pSTS activation (Kilts et al., 2003;LaBar et al., 2003;Sato et al., 2004;Fox et al., 2009a;Foley et al., 2012). In studies of face and body perception, the pSTS has also been associated with the processing of the biological motions of changeable stimulus components (Puce et al., 1998;Allison et al., 2000;Haxby et al., 2000;Grossman and Blake, 2002;Pelphrey et al., 2003). These biological motions of stimuli (e.g., an individual’s gaze or changes in facial muscles) apparently convey relatively complex social cues that are critical for adequate social communication and are therefore likely to evoke strong neural activation patterns.

### INDIVIDUAL DIFFERENCES IN EMOTION PROCESSING

When the individuals’ fMRI data were realigned based on the obtained behavioral data, certain brain areas demonstrated enhanced group-wise profiles for categorical/dimensional processing, whereas other regions exhibited no enhancement (**Figures 6** and **7**). In particular, the processing profiles of the right FFA and mPFC became more distinct, whereas the processing profile of the left amygdala became more obscure, and the processing profile of the left insula was enhanced in one dimension of the emotional space but obscured in the other dimension. These differences may reflect differences in the hierarchical organization of processing stages in the face-processing network. As discussed above, it is known that the FFA processes the psychological rather than the physical aspects of faces (Rotshtein et al., 2005;Fox et al., 2009b) and that the mPFC is activated when a subject perceives or attempts to regulate the effects of an emotionally evocative stimulus (Lane et al., 1997;Reiman et al., 1997;Ochsner et al., 2002;Phan et al., 2002). These findings suggest that the FFA and mPFC may participate in higher-order stages of face processing, implying that these areas may reflect explicit behavioral strategies. In contrast, the amygdala mediates implicit learning even for emotional stimuli that are not consciously perceived (Morris et al., 1998). The amygdala exhibits anatomically diverse connections to both lower- and higher-order brain areas and is therefore subject to both top-down and bottom-up modulations of its responses (Pessoa and Adolphs, 2010). Our implicit paradigm of facial emotion processing might involve reduced top-down modulation and enhanced bottom-up processing in the amygdala, resulting in amygdala activity that is significantly correlated with the physical changes of facial stimuli (as indicated by the “Stimulus-based” results in **Figure 6C**) but not the psychological changes of these stimuli (as indicated by the “Perception-based” results in **Figure 6C**). An anatomically and functionally graded representation of facial stimuli is thought to form within the structure of the insula, resulting in; a posterior-to-mid-to-anterior pattern for the integration of interoceptive information in this region (Craig, 2009). In particular, the integration of subjective (self-related) and objective (other-related) information in the anterior-/mid-insula may support our finding that there exists a relatively stable yet complex relationship between insula activity and the physical and psychological changes in facial stimuli, with changes in insula activity dependent on alterations in the dimensions of these stimuli (**Figures 6B** and **7B**).

The implicit hybrid processing that we observed might be achieved through the integrated function of separate neural loci. In particular, both types of processing involved in perceiving facial emotions may occur in the cortex for the “psychological” aspects of facial stimuli and in the subcortical/limbic system for the “physical” aspects of these stimuli; these processing mechanisms could function synergistically to produce the hybrid categorical/dimensional processing of facial emotion perceptions.

### FUTURE WORK

We examined the functional dissociation between the two fundamental dimensions of valence and arousal in the emotional space by creating two orthogonal continua of morphed stimuli that were parallel to these dimensions. Although we employed these morphed faces to generate inferences regarding dimensional and categorical perspectives (Fujimura et al., 2012), greater caution might be required before utilizing the morphed stimuli of this study as direct, independent representations of valence and arousal for drawing conclusions regarding neural representations.

(1) The affect grid ratings indicating that the happiness–fear continuum is categorical may simply reflect the fact that happiness is the most obvious positive emotion among the largely negative emotions that comprise the set of basic emotions (with the potential exception of surprise, which tends to be relatively neutral). This phenomenon could indirectly relate to the concept of the valence scale as bivariate scales indicating degrees of positivity and negativity (Cacioppo and Berntson, 1994), which may cause valence to be categorically distinguished more readily than arousal.
(2) Anger and disgust do not span arousal in a valence-neutral manner; instead, both emotions represent negative-valence forms of arousal. An independent measure of arousal would include both positive- and negative-valence expressions. In addition, it has been demonstrated that anger and disgust are structurally similar (Susskind and Anderson, 2008); thus, these two emotions might readily be perceived in a more similar fashion than the distinct opposites of fear and happiness. (However, the distinct categorical separation of disgust and anger may have been emphasized in the identification task of this study by the fact that only two options were offered.)

Thus, it might not be evident that the morphed-face stimuli of this study represent continua of valence and arousal. Instead, it may be more appropriate to clarify that these morphed-face stimuli represent indices of categorical vs. dimensional perception rather than clearly defined delineations. The definitive region-based coding of the valence and arousal dimensions and specific facial expression categories would require the use of a broader range of controlled stimuli.

## Conflict of Interest Statement

The authors declare that the research was conducted in the absence of any commercial or financial relationships that could be construed as a potential conflict of interest.
